# Progress in Medicine

**Published:** 1897-10-23

**Authors:** 


### Progress in Medicine.

### RHEUMATISM AND GOUT).

(*Continued from page* 27.)

An important examination of the various theories of the pathology of gout has been made by A. P. Luff[1] in his Goulstonian lectures. He argues that uric acid is not found normally in the blood of mammals or birds, and is in health produced only in the kidneys, probably by combination of urea with glycocine. If from any defect of the kidney it is not excreted, it is absorbed from the kidneys into the blood as a quadri-urate of sodium. This is gradually transformed into a biurate, which becomes at last deposited in a crystalline form in places where the circulation is weak or some injury has occurred. After deposition these crystals act as foreign bodies and irritate the surrounding tissues, though the dissolved urates may be harmless. Their presence in the blood in gout, then, is due to kidney failure; that is, probably to some affection of the convoluted tubules.

When uric acid is formed from nuclein in diseases where leucocytosis is present, it is rapidly eliminated by the kidneys before the change into the crystallisable biurate can take place. Thus kidney disease is probably a necessary forerunner of gout; but this failure of the kidneys is not always accompanied by albuminuria or dropsy. On the other hand, deposits have been found in the joints of 77 per cent. of cases of marked granular disease of the kidneys where gout had not been noticed during life. Luff concludes, as to the means we have for re-dissolving the sodium biurates in the joints, that the vegetable salts seem to have some power both in preventing and removing these deposits, but the degree of alkalinity of the blood has no influence. The saline constituents of meat actually decrease the solubility of these salts, but the action of wines in bringing on attacks of gout is probably due to their effect on the liver, and not to any acid contained in them.

Sir Willoughby Wade[2] points out that the tenderness round a gouty joint is chiefly along the course of the nerves, and not in the joint itself. The pains are of a neuralgic type. This neurosis is easily started by an injury, and kept up by the failure of recuperative power in a system poisoned by the gout. The uratic deposits in the joints seem to him to have comparatively little importance, for the pain may be very great in an acute attack where the deposits are very small, and the reverse in a chronic one where the deposits are very large. The nerve injury then is the essential lesion. If a sensory nerve is attacked we get pain and tenderness; if a vaso-motor one, redness and swelling; if a nutritive one, changes in the joint, one result of which may be a deposit of urates. Why one nerve is attacked in this case and another in that, may depend on the production of gout by various poisons allied to, but not identical with, uric acid.

Mordhurst[3] holds a view as to the deposition of uric acid, which is slightly different from Luff's. He considers that when the uric acid in the blood reaches a certain amount it is precipitated in a globular form, but if the alkalinity decreases, the globular urates change into acicular ones at a rate which varies inversely with the amount of sodium they contain. As long as the globular urates exist we have the symptoms of "rheumatism." As they vary in size from the most minute forms they may be quite invisible in the joints on post-mortem examination, but if the alkalinity of the blood is lessened they change into needles, pierce and destroy the tissues, and block up the lymphatics, causing the symptoms and the deposits found in gout. They may be changed back into the globular form in favourable circumstances, and even dissolve finally altogether and escape by the kidneys. Thus the distinction between rheumatism and gout is a matter of degree, if his views of crystallisation are correct. As a means of diagnosis between gout and rheumatoid arthritis, C. E. Nammack[4] remarks that the Röntgen rays show a splint-like deposit of urate of soda round the joints and tendons, while the ends of the bones are unchanged. In the second disease we may see exostoses formed at one edge of the articular surface, with absorption of the opposite side of the joint. F. W. Goodbody[5] investigated for the British Medical Association the source of the uric acid. He rendered the liver inactive, and found that some diminution of the nitrogen and urea in the urine then took place, though not so great as would be expected, while the uric acid rose in some cases and fell in others. When it was increased leucocytosis also occurred. If pilocarpin was injected to produce leucocytosis the uric acid increased for two or three hours, while the nitrogen, urea, and ammonia decreased. Thus, so far as these experiments go, they confirm the belief that uric acid is formed either in the kidneys or at times from the broken up white corpuscles. Haig's view that it is largely derived from the uric acid in the food consumed is opposed by Luff because of the unreliability of the method (Haycraft's) which Haig employed as the test of its presence in food, and because of its absence from the blood in healthy persons and animals. Nor is there a fixed relation between the excretion of uric acid and urea in health, as he supposed, for numerous experiments show that the ratio varies widely with the food and other factors while perfect health exists.

**Treatment.**—W. K. Sibley[6] reports a series of cases of gout treated by very hot dry air. The affected limb was kept in a receptacle containing air heated to something under 300 deg. for a space of 30 to 60 minutes. The treatment after a few applications rapidly dispelled the pain and swelling, and usually much sooner than under internal medication alone. In some instances the attack reappeared in a few days in another limb, from which it was quickly driven by the same treatment. French observers have noted a great increase in the excretion of uric acid, urea, and chlorides following the use of this apparatus. An exclusive red meat diet has been advocated by W. Armstrong[7] in certain cases of chronic gout, recurrent calculi, and amylaceous dyspepsia, which resist ordinary methods. It is, however, irksome, and requires great care, especially where disease of the heart or kidneys exists, and he thinks it is only applicable in some three or four per cent. of the cases. Similar improvement is seen in some instances under Haig's meat free dietary, and the results would thus appear to depend in part on not mixing meat and carbohydrate foods. It is of great importance not to allow any carbohydrate food in the first weeks of the course, which may last with advantage from one to three months. The patient drinks from three to five pints daily of hot water. One pint is taken an hour before each meal in sips, and the same quantity at bedtime. The food consists of pure lean meat minced, with the whites of two poached eggs if desired, and a little salt, pepper, mustard, and lemon juice. Coffee, weak tea, and, if necessary, a little whiskey may be given. A loss of flesh but an increase of activity is noticed, the pain and swelling of the joints soon diminish, and dyspeptic symptoms vanish. The only carbohydrate allowed is a small thin slice of toast with each meal until towards the end of the course of treatment. Levison's[8] diet for gout is framed to reduce the amount of nuclein broken up in the system. Hence he forbids liver and other internal meats, the yolk of eggs, alcohol, and coffee. To wash out the kidneys plenty of water and milk are ordered, but it is not desirable to make the urine too alkaline. Von Norden gives also calcium carbonate where gravel is formed, which is eliminated as a phosphate and reduces the amount of the monosodium phosphate in the urine. In this way the uric acid is kept in solution more easily. Grawitz finds that hot sand baths relieve the pains, and that very great benefit follows the application of a strong continuous current acting through a bath of lithium carbonate and chloride mixed, the negative pole being placed in a solution of common salt. H. C. Wood[9] believes that no single dietary is good, but that the individual idiosyncrasies should be carefully met. Exercise must be kept short of exhaustion. Massage and cycling are often beneficial, and hot baths should be frequently given. Colchicum, combined with ammonium and strontium salicylates, are valuable, and may be given, if necessary, with digitalis and strychnine. The hot-air treatment is not one that he finds of much use except where there are deposits in the tendons and outside the joints, as well as in strains and subacute rheumatism. Fawcett[10] tested the excretion of urea and uric acid in chronic gout in thirteen persons. The uric acid was well below the normal between the attacks, and somewhat higher, though still subnormal, during the acute stage. The increased acidity of the urine was not always accompanied with a fall of the excretion of uric acid, though the amount of urea varied closely with the acidity. Malfatti found that xanthin bases followed the same rule as uric acid in Fawcett's experiments. Rommel, too, denies that there is any increase in the excretion of alloxuric bodies in gout, but in cases of interstitial nephritis without gout found an increase of them, and still more of the uric acid.

[1] Lancet, March 27. [2] B. Med. Jour., Feb. 27. [3] Lancet, July 17. [4] Med. Record, Ap. 24. [5] B. Med. Jour., July 17. [6] Lancet, July 10. [7] Lancet, July 3. [8] Amer. J. Med. Sc., May. [9] N. Y. Med. Jour., Aug. 21. [10] Guy's Hosp. Reports.

